# A simple, yet novel hybrid‐dynamic conformal arc therapy planning via flattening filter‐free beam for lung stereotactic body radiotherapy

**DOI:** 10.1002/acm2.12868

**Published:** 2020-04-03

**Authors:** Damodar Pokhrel, Matthew Halfman, Lana Sanford

**Affiliations:** ^1^ Department of Radiation Medicine Medical Physics Graduate Program University of Kentucky Lexington KY USA

**Keywords:** FFF‐beam, hybrid‐DCA, lung SBRT, VMAT

## Abstract

**Purpose:**

Due to multiple beamlets in the delivery of highly modulated volumetric arc therapy (VMAT) plans, dose delivery uncertainties associated with small‐field dosimetry and interplay effects can be concerns in the treatment of mobile lung lesions using a single‐dose of stereotactic body radiotherapy (SBRT). Herein, we describe and compare a simple, yet clinically useful, hybrid 3D‐dynamic conformal arc (h‐DCA) planning technique using flattening filter‐free (FFF) beams to minimize these effects.

**Materials and Methods:**

Fifteen consecutive solitary early‐stage I‐II non‐small‐cell lung cancer (NSCLC) patients who underwent a single‐dose of 30 Gy using 3–6 non‐coplanar VMAT arcs with 6X‐FFF beams in our clinic. These patients’ plans were re‐planned using a non‐coplanar hybrid technique with 2–3 differentially‐weighted partial dynamic conformal arcs (DCA) plus 4–6 static beams. About 60–70% of the total beam weight was given to the DCA and the rest was distributed among the static beams to maximize the tumor coverage and spare the organs‐at‐risk (OAR). The clinical VMAT and h‐DCA plans were compared via RTOG‐0915 protocol for conformity and dose to OAR. Additionally, delivery efficiency, accuracy, and overall h‐DCA planning time were recorded.

**Results:**

All plans met RTOG‐0915 requirements. Comparison with clinical VMAT plans h‐DAC gave better target coverage with a higher dose to the tumor and exhibited statistically insignificance differences in gradient index, D_2cm_, gradient distance and OAR doses with the exception of maximal dose to skin (*P* = 0.015). For h‐DCA plans, higher values of tumor heterogeneity and tumor maximum, minimum and mean doses were observed and were 10%, 2.8, 1.0, and 2.0 Gy, on average, respectively, compared to the clinical VMAT plans. Average beam on time was reduced by a factor of 1.51. Overall treatment planning time for h‐DCA was about an hour.

**Conclusion:**

Due to no beam modulation through the target, h‐DCA plans avoid small‐field dosimetry and MLC interplay effects and resulting in enhanced target coverage by improving tumor dose (characteristic of FFF‐beam). The h‐DCA simplifies treatment planning and beam on time significantly compared to clinical VMAT plans. Additionally, h‐DCA allows for the real time target verification and eliminates patient‐specific VMAT quality assurance; potentially offering cost‐effective, same or next day SBRT treatments. Moreover, this technique can be easily adopted to other disease sites and small clinics with less extensive physics or machine support.

## 1 Introduction

With the development of more precise and accurate treatment delivery, stereotactic body radiation therapy (SBRT) treatment of medically inoperable early‐stage non‐small‐cell lung cancer (NSCLC) patients shows higher tumor local‐control rate and minimal treatment‐related toxicity.^1^, ^2^, ^3^, ^4^, ^5^ For the selected peripherally located NSCLC patients; single‐dose of SBRT has become a curative treatment option as shown by the randomized trials.^6^, ^7^, ^8^, ^9^, ^10^, ^11^, ^12^, ^13^ For instance, Videtic and colleagues^7^ compared 2 single‐fraction SBRT dosing schemes of 30 and 34 Gy for 80 medically inoperable early stage‐I NSCLC patients. Both treatment schedules provided similar tumor local‐control and overall survival rates with minimal pulmonary toxicity. Thus, a stereotactic, single‐dose of 30 Gy is an equally effective treatment for the selected NSCLC patients and it was radially adopted for patients treatment in our clinic. Recently, there has been growing interest in the clinical use of flattening filter free (FFF) beams to deliver lung SBRT treatment.^14^, ^15^, ^16^, ^17^, ^18^ FFF‐beams have much higher dose rates compared to traditional flattened‐beams that use flattening filters (FF). FFF beams can reduce beam on time (specifically beneficial for single large dose treatment), resulting in better patient comfort and reducing dose delivery uncertainty due to less intrafraction motion error and can potentially reduce out‐of‐field dose with less head scatter and electron contamination.^16^

Combining FFF‐beams with volumetric modulated arc therapy (VMAT)^18^, ^19^resulted in greater treatment efficiency for complex lung SBRT plans compared to historically used plans with 8–15 non‐coplanar fixed fields or several coplanar dynamic conformal arcs (DCA) with flattened‐beams.^19^, ^20^, ^21^The same results were observed when compared to linac‐based intensity‐modulated radiation therapy (IMRT), VMAT plans, helical TomoTherapy or optimized robotic CyberKnife plans (showing significant increases in SBRT treatment times).^22^, ^23^, ^24^, ^25^However, for single‐dose lung SBRT treatments, highly modulated VMAT plans are highly susceptible to delivery uncertainties due to small‐field dosimetry errors^26^and interplay effects^27^ due to multileaf collimator (MLC) modulation of multiple beamlets as a function of lung tumor motion.

Coupled with DCA, FFF‐beams allow for faster delivery of lung SBRT treatments with a steep dose fall‐off outside the target. Many researchers have studied the use of VMAT with FFF beams, but not much has been written yet on the use of DCA and FFF beams. Currently, in our clinic we use non‐coplanar VMAT lung SBRT plans with 3–6 partial arcs and 6 MV‐FFF (1400 MU/min) beams. However, often times there is concern about treating a moving target with a highly modulated beam as mentioned before. Additionally, delivering VMAT plan requires additional commissioning effort, potentially a higher degree of quality assurance (QA) and testing of Linac chain and tighter Linac tolerances due to smaller fields and variation in dose rate with simultaneous gantry and MLC movement. Because of that, sometimes‐delivering VMAT plan would be difficult with the older Linac. Furthermore, depending on the dose algorithm used there may be concerns over the accuracy of the calculation for small‐field dosimetry in areas of tumor‐tissue interfaces. DCA with FFF beams allow the user to take full advantage of the high dose rate with a decrease in the overall monitor units (MU) to deliver SBRT treatments in under a few minutes.

To address these issues we have designed a novel, yet simple hybrid‐DCA (h‐DCA) therapy planning technique that can reproduce lung SBRT plans similar to clinical VMAT plans in compliance with the RTOG‐0915 requirements.^6^ Our h‐DCA plans used a non‐coplanar hybrid technique with 2–3 differentially weighted partial DCAs (similar to those used by VMAT plans) plus 4–6 static beams depending upon tumor size and location on a per‐patient basis. About 60–70% of the beam‐weight was given to the DCA and the rest was distributed among the static beams to maximize the target coverage and minimize the dose to the organs‐at‐risk (OAR). Our h‐DCA provides highly conformal dose distributions by delivering doses with MLC dynamically conforming to the beam’s‐eye‐view (BEV) projections of the target and steers isodose distributions by using a few static‐beams. In contrast, VMAT delivers the optimized dose distribution by using many small beamlet‐based intensity modulations using a combination of several separated MLC segments per arc. Our h‐DCA plans are quicker to plan and deliver the lung SBRT treatment. Even though h‐DCA does not use intensity‐modulated beams, it still generates highly conformal radiosurgical dose distributions and satisfies the conformity and OAR requirements of the lung SBRT protocol. The h‐DCA plans could potentially minimize small‐field dosimetry errors and MLC interplay effects.

In this report, we describe a novel method and compare this simple, yet clinically useful 3D‐hybrid planning technique for single‐dose (30 Gy) SBRT treatments of the peripheral lung lesions. In addition, the delivery efficiency and overall planning time of the h‐DCA were estimated.

## 2 Materials and methods

### 2.A Patient characteristics

After obtaining an institutional review board approval from our institution, fifteen consecutive Stage I–II NSCLC patients with peripherally located tumors who underwent single‐dose lung SBRT treatments (30 Gy) were included in this study. In our clinic, only selected patients with isolated tumor located in the middle of the lungs (either left or right) where there are no major critical structures around the tumor qualify for a single‐dose (30 Gy) of lung SBRT treatment. For other lung lesions that are near the major critical structures such as spinal cord, heart or rib we use risk adopted prescriptions either 54 Gy in 3 fractions, 48 Gy in 4 fractions or 50 Gy in 5 fractions dosing schemata, depending upon the plan quality and treating physician preferences.

### 2.B Imaging and target definition

All patients were immobilized using Body Pro‐Lok^TM^ platform (CIVCO system, Orange City, IA, USA) in the supine position with their arms above their head using an armrest and abdominal compression. The free‐breathing planning 3D‐CT simulation was acquired on a GE Lightspeed 16 slice CT scanner (General Electric Medical Systems, Waukesha, WI, USA) with 512 × 512 pixels at 2.5 mm slice thickness in the axial helical mode. Following the 3D‐CT scan, all patients underwent a respiration‐correlated 4D‐CT scan using the Varian RPM System (version 1.7) in the same position. The 4D‐CT images were reconstructed in 10 equally spaced phase bins using an Advantage 4D Workstation (General Electric Medical Systems, San Francisco, CA, USA), where the maximum intensity projection (MIP) images were generated. The regular 3D CT and the MIP images were imported into Eclipse TPS (Version 13.6, Varian Medical Systems, Palo Alto, CA, USA) and co‐registered for target delineation. Gross tumor volume (GTV) = internal target volume (ITV) was delineated on the 3D‐CT images with reference to the MIP images. The planning target volume (PTV) was generated by adding a uniform 5 mm margin around the ITV per RTOG‐0915 guidelines.^6^The relevant critical structures included bilateral lungs excluding the ITV (healthy lung), spinal cord, ribs, heart, big vessels, esophagus, and skin.

The tumor characteristics are summarized in Table 1 including tumor size and location. The average ITV derived from 4D‐CT scan was 3.2 ± 4.6 cc (range 0.2–13.5 cc). The mean PTV was 14.7 ± 13.0 cc (range 4.3–41.1 cc), corresponding to an average tumor diameter of 2.8 ± 0.8 cm (range 2.0–4.22 cm).

**Table 1:** Characteristics of lung SBRT patients included in this study. Prescription dose was 30 Gy in 1 fraction

Patient no.	Tumor location	ITV (cc)	PTV (cc)	PTV diameter, *d* (cm)	Healthy lung volume (cc)
1	Left lower lobe	0.2	5.0	2.11	5088.2
2	Left lower lobe	0.33	5.1	2.12	4847.1
3	Left upper lobe	0.7	6.4	2.28	2390.0
4	Right lower lobe	0.75	8.2	2.48	2989.8
5	Left upper lobe	10.1	41.1	4.22	2885.3
6	Right lower lobe	1.1	10.7	2.71	6975.9
7	Left upper lobe	0.3	4.3	2.00	2636.0
8	Right lower lobe	13.6	37.5	4.09	4070.9
9	Left upper lobe	0.8	5.2	2.13	4069.2
10	Left upper lobe	1.4	11.0	2.73	2709.2
11	Left upper lobe	2.1	14.8	3.01	3692.8
12	Right lower lobe	2.5	14.4	2.99	5967.7
13	Left upper lobe	0.5	5.8	2.21	2352.9
14	Left lower lobe	2.0	13.3	2.91	2327.9
15	Right upper lobe	12.2	37.6	4.10	5109.0

ITV, internal target volume; PTV, planning target volume.

### 2.C Clinical VMAT plans

For the fifteen consecutive patients, clinically optimal VMAT‐SBRT plans were generated in Eclipse TPS using 3–6 (mean, 4) partial non‐coplanar arcs (with ±5–10° couch kicks) for a Truebeam linear accelerator (Varian Palo Alto, CA, USA) consisting of standard millennium MLC and 6MV‐FFF (1400MU/min) beams. The isocenter was placed at the geometric center of the PTV. These partial non‐coplanar arcs had an arc length of approximately 200–220°, and collimator angles (between 30° and 135°) were manually optimized to reduce the MLC tongue‐and‐groove dose leakage throughout the arc rotation on a per‐patient basis. Jaw tracking option was used during plan optimization to further minimize out‐of‐field dose leakage. The prescription dose was 30 Gy in 1 fraction to the PTV while covering at least 95% of the PTV with prescription dose and ensuring that all hot spots (between 120% and 130%) fall within the ITV. All clinical treatment plans were calculated using Eclipse TPS with the advanced Acuros‐XB (Version 13.6) algorithm 28, 29, 30, 31, 32 on the 3D‐CT images for heterogeneity corrections with 2.5 mm × 2.5 mm × 2.5 mm calculation dose grid‐size (CGS) and the photon optimizer (PO) MLC algorithm. The dose to medium reporting mode was used, and the planning objectives followed RTOG‐0915 requirements (Arm 1).^6^

### 2.D Quality assurance and treatment delivery

Before delivering each VMAT‐SBRT plan, a daily QA check on kilovoltage to megavoltage imaging isocenter coincidence was performed, including IsoCal measurement for the precise and accurate target localization. Our IsoCal localization accuracy for Truebeam was <0.5 mm. All the QA procedures including patient‐specific QA were in compliance for SBRT treatment delivery.^5^Our Octavius 4D (PTW, Freiburg, Germany) phantom (with an Octavius 1500 detector array insert) QA pass rate was 97.6% ± 2.7%, on average, for 3%/3 mm criteria. All patients were treated in our clinic with cone‐beam CT‐guided procedure on our Truebeam. On the treatment day, patient set up prior to single‐dose lung SBRT was performed using an in‐house SBRT/IGRT protocol;^6^ by co‐registering the pretreatment conebeam CT with the planning CT scan. Image registration was performed automatically based on the region of interest bony landmark, followed by manual refining performed by the treating physician to ensure that the tumor was registered within the ITV contoured on the planning CT. The patient position was re‐positioned for 6 degrees of freedom (6‐DOF) couch corrections according to the results of tumor soft tissue registration and the treatment was delivered. The 6‐DOF couch correction parameters were within the limits of our departmental lung SBRT protocol guidelines for all patients. The patient set up, tumor matching, and treatment delivery were monitored and verified by the treating physician and physicist.

### 2.E Hybrid‐DCA plans

For comparison, the standard clinical VMAT‐SBRT plans were re‐planned in Eclipse using a combination of 2–3 non‐coplanar differentially‐weighted partial DCA (similar to those clinical VMAT arcs) plus 4‐6 static beams (called h‐DAC plans) using 6X‐FFF beams. Choice of static‐beams injection depended on the tumor location. For instance, for right lung lesions a few static‐beams can be added between the gantry angles 350 to 180‐degrees separated by 30 to 40‐degrees gantry spacing. A similar approach was applied for the left lung lesions. For those arcs and static fields, optimal collimator rotations were used to minimize the tongue and groove effect for each patient. For the DCA(s), 2 mm MLC margins all around the PTV were automatically generated and maintained dynamically around the target during arc rotation. MLC margins of 1 mm in the lateral, anterior and posterior directions and 3 mm in the cranio‐caudal directions were used for the static beams. About 60–70% of the total beam weight was given to the DCA(s) and the rest was distributed differentially among the static beams to maximize the target coverage while minimizing the dose to OAR. This novel planning technique provides highly conformal dose distributions by delivering doses with MLC dynamically conforming to the BEV projections of the target and steers isodose distributions by using a few static‐beams. It provided both appropriate target coverage and tight conformity indices with plan renormalization. A 1 cm thick spherical ring structure located 2 cm away from the PTV wall in all directions (D_2cm_) was generated and used for dose steering (static gantry angles, spacing, and weight can be adjusted as needed) to ensure high conformity and minimal intermediate dose‐spillage per RTOG‐0915 requirements. Dose was calculated using the advanced Acuros‐based algorithm with identical CGS as clinical VMAT plan described earlier. However, some plans would be tuned further by manually tweaking the MLC margins on the static beams, applying negative margins to the MLC, changing beam‐weighting and renormalizing the plan until the plan quality met the requirements set by RTOG for acceptable target coverage and dose to OAR.

### 2.E Evaluation of dose distribution

The original clinical VMAT and re‐optimized h‐DCA plans were compared through RTOG‐0915 protocol compliance; target conformity (CI), heterogeneity index (HI), gradient index (GI) and dose to OAR. Additionally, delivery efficiency and overall 3D‐planning time were recorded. The dose volume histograms (DVHs) of all treatment plans were evaluated for the following RTOG‐0915 high and intermediate dose spillage dose parameters:^6^

- Conformity index, CI: ratio of prescription isodose volume to the PTV. CI less than 1.2 is desirable; CI = 1.2 to 1.5 is acceptable with minor deviations.
- Gradient index, GI: ratio of 50% prescription isodose volume to the PTV. GI has to be smaller than 3‐6, depending on the PTV.
- Maximum dose at any point 2 cm away from the PTV margin in any direction, D_2cm_: D_2cm_ has to be smaller than 50‐70%, depending on the PTV.
- Percentage of normal lung receiving dose equal to 20 Gy or more, V_20_: V_20_ should be less than 10% per protocol, V_20_ less than 15% is acceptable with minor deviations.
- Heterogeneity index, HI: HI = Dmax/prescribed dose was used to evaluate the dose heterogeneity within the PTV.
- Gradient distance, GD: GD is the average distance from 100% prescribed dose to 50% prescribed dose, which indicates how sharp the dose falls off. The GD is used to evaluate dose sparing to normal lung volume. The smaller the value of GD, the faster the dose fall‐off.
- Total number of MU.
- Modulation factor, MF: ratio of total number of MU to the prescription dose in cGy.
- Beam‐on time (BOT): BOT was recorded during phantom QA measurement at the machine.

Furthermore, all clinical VMAT and h‐DCA plans were evaluated for the relative volume of normal lung receiving 5 and 10 Gy, the mean lung dose (MLD) and the maximum dose received by 1000 cc of lungs. Dose to the spinal cord (maximum and 0.35 cc), heart (maximum and 15 cc), and esophagus (maximum and 3 cc) were analyzed. Since these were peripheral lung lesions, the doses to ribs (maximum and 1 cc) and skin (maximum and 10 cc) were also evaluated. The mean and standard deviation for each dose metric was compared using two‐tailed paired t‐tests (using an upper bound *P*‐value of <0.05) for the clinical VMAT vs h‐DCA plans for all dosimetric parameters, target coverage, dose tolerances to OAR and treatment delivery parameters. Dose limits for maximum doses to spinal cord <14.0 Gy, heart <22.0 Gy, esophagus <15.4 Gy, maximum dose and dose to 1 cc of ribs, <30.0 Gy and <22.0 Gy, maximum dose and 10 cc of skin <26.0 Gy and <23.0 Gy were used per single‐dose SBRT protocol recommendations, respectively.

## 3 Results

### 3.A Target coverage

All plans met RTOG 0915 requirements. Compared to clinical VMAT plans, h‐DCA plans showed similar conformity and target coverage, yet better tumor heterogeneity and exhibited no statistical significance in intermediate dose‐spillage (GI, D_2cm_ and GD, see Table 2). Although the CI with h‐DCA plans were slightly higher than clinical VMAT plan, providing a little leeway could be beneficial for target coverage while respecting the OAR dose (for mobile lung lesions). h‐DCA plans showed a higher dose to the ITV (corresponding to higher values of HI, unique characteristics of the SBRT plan). The maximum, minimum and mean doses to the tumor (ITV) were higher by about 2.8, 1.0, and 2.0 Gy, respectively, on average 10% higher compared to clinical VMAT plans with no additional cost. This is a characteristic behavior of the dose profile of the FFF‐beams used; delivering a higher dose to the tumor center as desired in the SBRT treatment.

**Table 2:** Evaluation of target coverage for all 15‐lung SBRT patients for both plans. Prescription was 30 Gy in 1 fraction. Mean ± SD (range) was reported

Target volume	Parameters	Clinical VMAT	Hybrid‐DCA	*P*‐value
PTV	CI	1.06 ± 0.07 (0.97–1.24)	1.13 ± 0.04 (1.09–1.26)	**<0.001**
	HI	1.21 ± 0.1 (1.1–1.29)	1.29 ± 0.1 (1.21–1.39)	**0.001**
	GI	5.34 ± 1.11 (3.81–7.23)	5.10 ± 0.71 (3.86–6.15)	n. s.
	D_2cm_ (%)	49.4 ± 4.7 (37.8–55.4)	51.11 ± 4.7 (44.2–60.3)	n. s.
	GD (cm)	1.03 ± 0.2 (0.77–1.37)	1.02 ± 0.2 (0.84–1.33)	n. s.
ITV	D_max_ (Gy)	35.9 ± 1.5 (33.07–37.68)	38.7 ± 2.1 (36.35–41.90)	**0.001**
	D_min_ (Gy)	32.3 ± 1.2 (28.85–33.85)	33.2 ± 1.6 (29.61–36.20)	**0.048**
	D_mean_ (Gy)	34.5 ± 1.1 (32.45–36.31)	36.5 ± 1.7 (34.52–39.68)	**0.001**

Statistically significant *P*‐values are highlighted in bold.

ITV, internal target volume; PTV, planning target volume; n. s., not statistically significant.

**Fig. 1:**
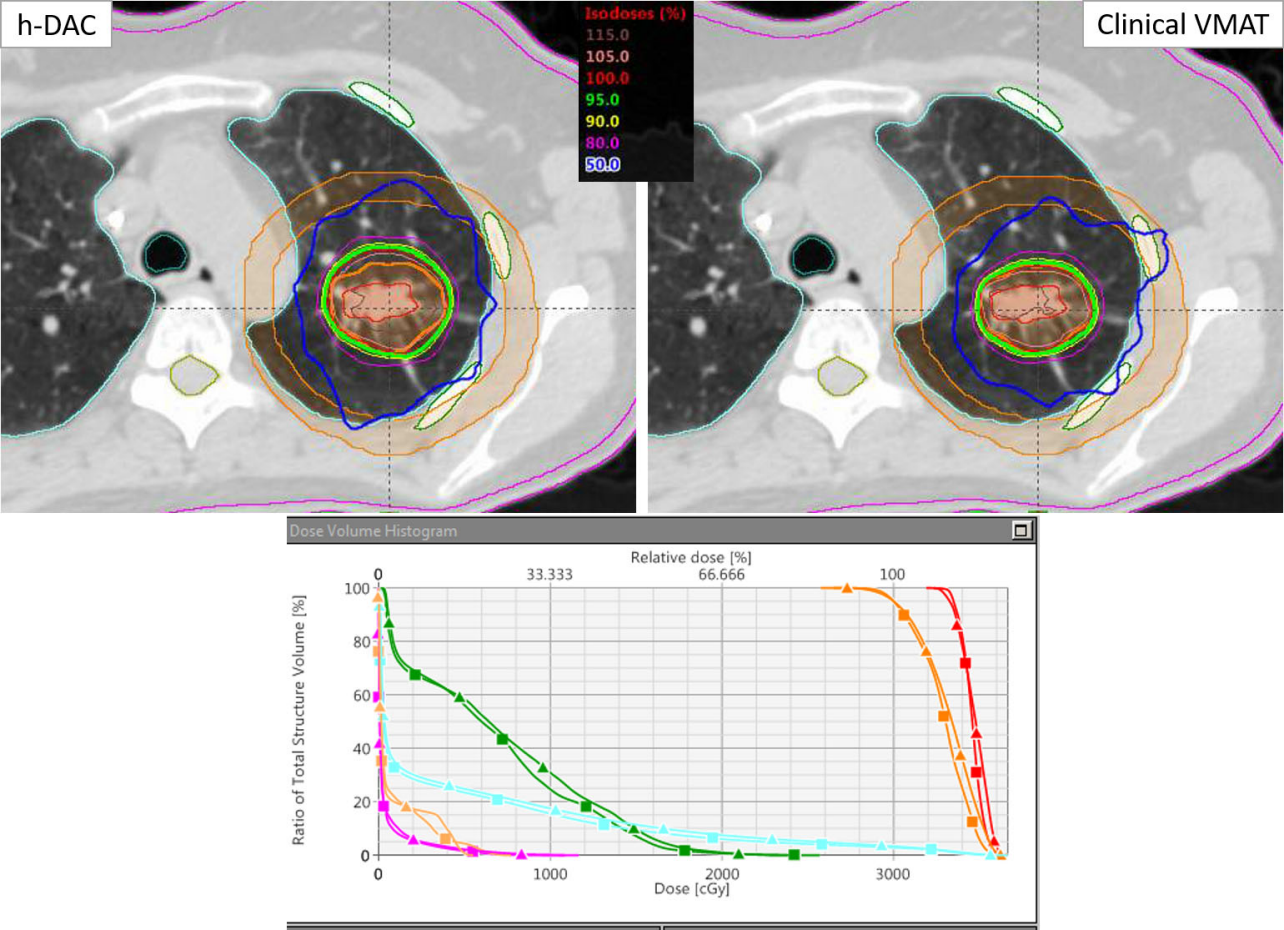
Comparison of h‐DCA vs a clinical volumetric modulated arc therapy (VMAT) plan for the example case described above. The upper panel shows radiosurgical‐isodose distributions for the h‐DCA and clinical VMAT plan. Similar, CI, HI, GI, D2cm, GD and V_20Gy_ were obtained. A few critical structures shown were ribs, cord, normal lung as well as D2cm ring. The lower panel shows the DVH comparison for both plans. Triangle shows the h‐DCA and square shows the clinical VMAT plan (red, ITV; Orange, PTV; green, ribs; light blue, normal lung; and pink, skin). Identical target coverage and similar OAR sparing were achieved with h‐DCA, but with a shorter treatment time and more accurate treatment delivery

No dosimetric differences in terms of dose to OAR were observed. Both plans achieved the RTOG‐0915 protocol compliance and were clinically acceptable for stereotactic treatment. Figure 1 shows an example case of radiosurgical dose distributions in axial view through the isocenter plane for example lung SBRT patient planned via clinical VMAT (top right panel) and h‐DCA (top left panel). DVH parameters (bottom panel) are shown for the target coverage and OAR doses for clinical VMAT vs h‐DCA plan; showing dosimetrically equivalent plans. A clinically similar SBRT plan was reproduced using a forward planning approach with h‐DCA compared to the inversely optimized VMAT plan. The PTV was 41.1 cc (4.2 cm diameter). This is a relatively large tumor size in this cohort and located in the left upper lobe. In this case, the CI, HI, GI, D_2cm_, GD, and normal lung V_20Gy_ were 1.14 vs. 0.97, 1.22 vs. 1.20, 4.09 vs. 3.82, 60.3% vs. 59.1%, 1.33 cm vs.. 1.29 cm and 2.14% vs. 1.45%, h‐DCA vs. clinical VMAT plan, respectively.

### 3.B Dose to OAR and delivery efficiency

The dosimetric differences (mean and standard deviation) between clinical VMAT and h‐DCA plans for the OAR (spinal cord, heart, esophagus, ribs, skin, and normal lung) and delivery parameters including the phantom QA results are listed in Table 3. One case whose tumor was abutting rib had slightly higher than protocol suggested rib dose on both plans. Although, statistically insignificant differences (*P*‐value >0.05) were found for all the evaluated dosimetric parameters except for maximum dose to skin, which increased slightly with h‐DCA plans compared to clinical VMAT plans (*P* = 0.015, highlighted in bold). However, for the maximal dose to skin, the absolute differences were typically less than 1.0 Gy and well below SBRT protocol guidelines. Therefore, that difference is not expected to be clinically significant. If needed, skin dose can also be managed by adding one more static field in the h‐DCA plan. Maximum dose to 1000 cc of lung was also similar for both plans (not shown here). However, comparison of treatment delivery parameters (total MU and BOT) significantly favored the h‐DCA plans (see Table 3); the average BOT was improved by a factor of 1.51.

**Table 3:** Evaluation of dose to OAR and treatment delivery efficiency for all 15‐lung SBRS patients for both plans. Prescription was 30 Gy in 1 fraction. Mean ± SD (range) was reported

OAR	Parameters	Clinical VMAT	Hybrid‐DCA	*p*‐value
Spinal cord	D_max_ (Gy)	3.4 ± 2.5 (0.5–6.53)	3.3 ± 2.6 (0.5–7.68)	n. s.
	D_0.35cc_ (Gy)	3.1 ± 2.4 (0.2–6.91)	3.1 ± 2.4 (0.4–7.01)	n. s.
Heart	D_max_ (Gy)	7.7 ± 4.7 (0.15–17.62)	8.3 ± 4.6 (0.4–17.27)	n. s.
	D_15cc_ (Gy)	3.7 ± 2.3 (0.1–9.0)	4.3 ± 2.6 (0.14–9.2)	n. s.
Esophagus	D_max_ (Gy)	4.1 ± 2.1 (0.2–7.49)	4.1 ± 2.2 (0.14–6.99)	n. s.
	D_3cc_ (Gy)	2.1 ± 1.4 (0.1–4.49)	2.6 ± 1.9 (0.1–5.69)	n. s.
Skin	D_max_ (Gy)	9.1 ± 2.6 (5.46–14.47)	9.9 ± 1.9 (7.89–13.97)	**0.015**
	D_10cc_ (Gy)	4.9 ± 1.5 (2.9–7.92)	5.2 ± 2.1 (0.7–8.95)	n. s.
Ribs	D_max_ (Gy)	21.6 ± 6.8 (11.4–31.51)	20.6 ± 6.4 (12.0–31.69)	n. s.
	D_1cc_ (Gy)	16.5 ± 4.3 (9.4–24.01)	16.0 ± 4.0 (9.6–23.63)	n. s.
Healthy lung	V_20Gy_ (%)	0.64 ± 0.4 (0.14–1.45)	0.73 ± 0.6 (0.19–2.14)	n. s.
	V_10Gy_ (%)	2.9 ± 1.9 (0.6–6.48)	3.1 ± 2.1 (0.79–6.87)	n. s.
	V_5Gy_ (%)	7.1 ± 3.9 (1.7–14.9)	7.2 ± 4.1 (1.8–15.64)	n. s.
	MLD (Gy)	1.25 ± 0.6 (0.53–2.33)	1.29 ± 0.6 (0.56–2.39)	n. s.
Delivery parameters	Total MU	8974 ± 1902 (7246–14684)	5949 ± 908 (4360–7673)	**<0.001**
	MF	3.0 ± 0.63 (2.2–4.9)	—	—
	BOT (min)	6.41 ± 1.36 (4.6–10.49)	4.25 ± 0.65 (3.11–5.48)	**<0.001**
	QA pass rate (%) 2%/2mm γ criteria	90.5 ± 7.7 (83.0–93.3)	98.6 ± 1.4 (96.5–100.0)	**<0.001**

Statistically significant *P*‐values are highlighted in bold.

ITV, internal target volume; PTV, planning target volume; n. s., not statistically significant.

**Fig. 2:**
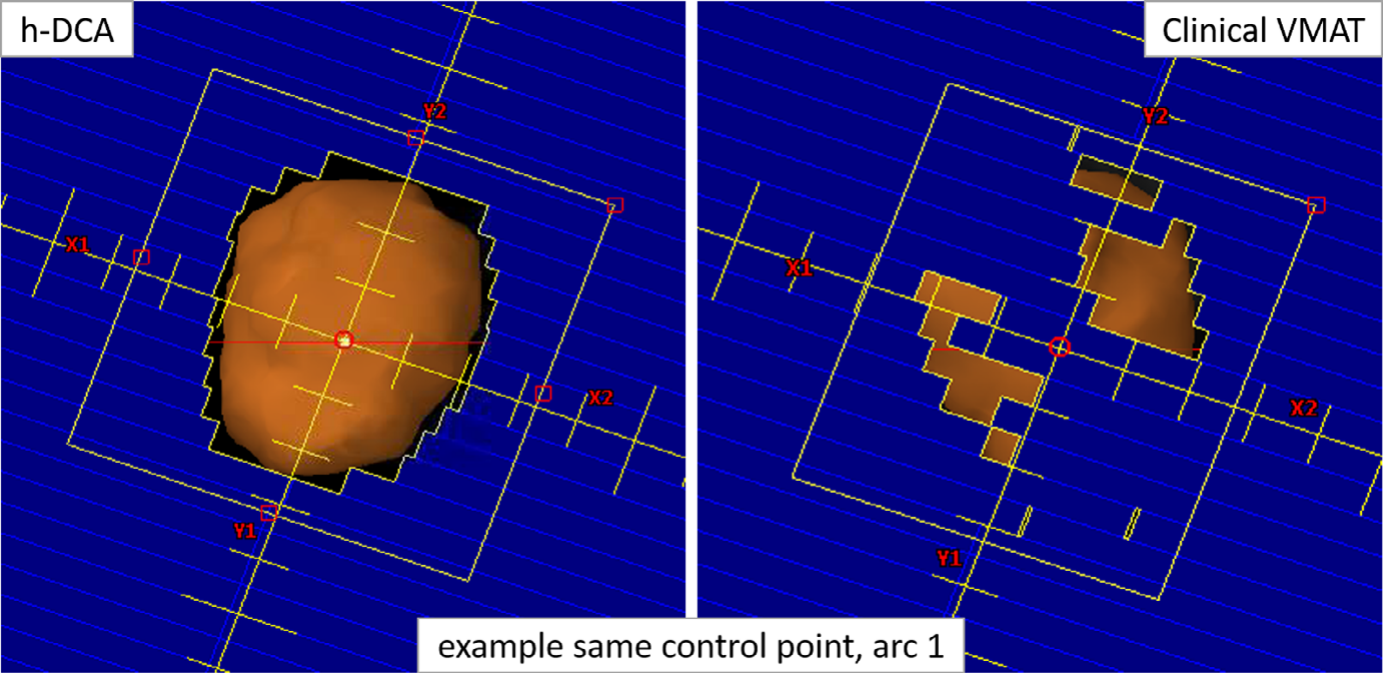
Comparison of a selected MLC control point (one control point for arc 1 on each plan) between the h‐DCA and clinical volumetric modulated arc therapy (VMAT) plans (same patient shown in Fig. 1). The h‐DCA MLC pattern (left panel) conforms to the PTV (orange) while the majority of the PTV is under the MLC block, due to MLC modulation, in the clinical VMAT plan (right panel). Although both plans provided similar target coverage and dose to OAR, h‐DCA plans delivered treatments faster and potentially more accurately due to the lack of MLC modulation across the target. This is believed to potentially minimize the concerns of small‐field dosimetry and MLC interplay effects

The improvement of treatment delivery efficiency is directly associated with the h‐DCA planning technique (forward planning approach) with no beam modulation through the PTV as shown in Fig. 2. In addition to the uncertainty of modeling of small‐field dosimetry, there is a potential concern that the interplay effects between the dynamic MLC modulation and tumor motion can degrade the delivery accuracy compared to the calculated values based on static plans. This study does not quantify the variation of the delivered dose due to the tumor motion when calculating the dose distributions. However, h‐DCA always fit the control point shape to the projection of the target at each gantry angle (see Fig. 2), thus minimizing MLC modulation and reducing the interplay effects. Therefore, the main benefit of the h‐DCA plan is the reduced MU and BOT required to deliver the same prescription dose. There is no beam modulation across the target volume with h‐DCA plan; therefore, we did not calculate MF for h‐DCA plans (see Table 3). Although, for the clinical VMAT plans the MF factor was up to 4.9 (average 3.0 ± 0.63) suggesting highly modulated treatment deliveries. The dose delivery accuracy of these clinical VMAT plans, the corresponding h‐DCA plans were 90.5% ± 7.7% and 98.6% ± 1.4%, on average respectively with 2%/2 mm gamma passing rate criteria while using an Octavius QA phantom measurement –suggesting that significant dose deviation can be seen with highly modulated clinical VMAT plans compared to h‐DCA plans.

**Fig. 3:**
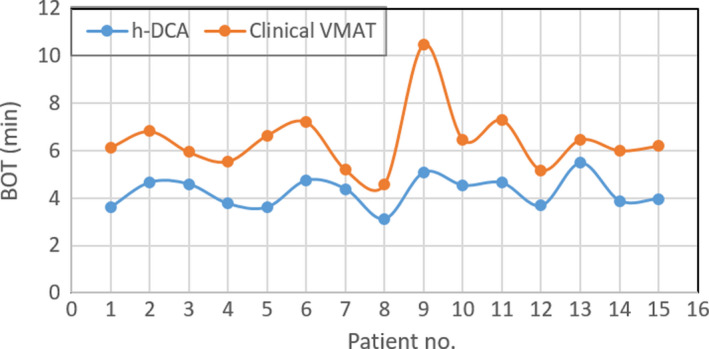
Total BOT on a per‐patient basis, for all 15 lung SBRT patients treated with a single‐dose of 30 Gy: mean value of BOT was 4.25 ± 0.65 min (with h‐DCA) compared to 6.41 ± 1.36 min (with clinical volumetric modulated arc therapy); showing a reduction of BOT by a factor of approximately 1.51 on average, and systematically on all patient plans

Comparison of BOT on a per‐patient basis is shown in Fig. 3. It has been observed that BOT was systematically lower for all patients and improved by a factor of 1.51, on average, when utilizing the h‐DCA plans. The lower BOT will reduce the time the patient is on the table, thus improving patient comfort and potentially reducing errors due to intra‐fraction tumor motion as well.

## 4 Discussion

A novel hybrid forward planning approach is presented for rapid and RTOG‐0915 compliant treatment planning and delivery using h‐DCA plans for a single‐dose of 30 Gy lung SBRT treatments. h‐DCA SBRT plans involved a non‐coplanar hybrid technique with 2–3 differentially weighted partial DCAs plus 4–6 static beams with specific collimator angles to minimize dose leakage. As described earlier, nearly 60–70% of the total beam weight was assigned to the DCA(s) and the rest of was unevenly distributed among the static beams to maximize the tumor dose and minimize the dose to OAR. The h‐DCA SBRT plans were highly conformal (similar to clinical VMAT plans) and achieved similar target coverage (see Table 2) compared to clinical VMAT plans. For all patients, the h‐DCA plans met RTOG dosimetric compliance criteria including normal lung V_20Gy_, V_10Gy_, V_5Gy_ and were similar to clinical VMAT plans. The other OAR (spinal cord, heart, esophagus, ribs, and skin, see Table 3) were well below protocol dose guidelines. The h‐DCA plans required less MU to deliver the same total prescribed dose due to no beam modulation across the target. Therefore the BOT was reduced (average BOT 4.3 min) demonstrating the efficiency of h‐DCA plans for a single‐dose lung SBRT treatments in selected patients. With h‐DCA plans, the BOT can be reduced 60–70% compared with clinical VMAT (average BOT 6.41 min). Furthermore, the average treatment planning time for h‐DCA plan was about an hour for an experienced physicist suggesting that the possibility of the same or next day of SBRT treatment to lung lesions.

A study by Dong *et al*.^33^ compared 4π plans with 7–9 static‐beam IMRT plans and VMAT plans prescribed to 50 Gy in 4 fractions for 12 centrally located lung tumors patients. The 4π algorithm used up to 30 optimized coplanar and non‐coplanar fields. In their study, it was concluded that compared to IMRT and VMAT, the 4π plans gave significantly and consistently better target coverage and critical OAR sparing. However, the 4π treatment delivery time was not reported. We believe that delivering 30 non‐coplanar fields to treat lung SBRT patients would be clinically impractical for current Linac/clinic workflows. In contrast, utilizing our h‐DCA technique with 6 MV‐FFF beam can deliver much faster and effective curative single‐dose SBRT treatments for these selected NSCLC patients.

The change in respiratory patterns between the CT simulation and the time of treatment has been studied in the past.^34^, ^35^, ^36^, ^37^Although, it has been reported that there were only small changes (within ±3 mm) due to intrafractional and interfractional motion in lung SBRT treatments, the mean patient set up time from tumor localization to the end of treatment cone beam CT scan was about 40 min.^36^ It was suggested that an isotropic 5 mm PTV margin around the ITV was sufficient to address these potential motion errors. Furthermore, the interplay effect between the MLC modulation and gantry rotation as a function of tumor motion could introduce dose blurring on highly modulated VMAT plans, which can be of another concern for a single‐dose lung SBRT treatment.^27^ In this study, the average beam on time was 4.3 min for single‐fraction SBRT treatment with h‐DCA and 6X‐FFF beam; decreasing the variation in dose delivery due to coughing or pain and making geographic miss less likely by improving the patient stability on the table. In addition, delivery of h‐DCA with FFF‐beams can minimize the major concerns of small‐field dosimetry errors and potentially reducing MLC interplay effects (with no MLC modulation through the target) that persists with highly modulated VMAT plans–demonstrated by a high QA pass rates on phantom measurement.

Other possible worries for lung SBRT treatments are low dose‐spillage in the chest wall and ribs;^38^, ^39^, ^40^ normal lung (V_20Gy_, V_10Gy_ and V_5Gy_)^41^, ^42^ and dose to skin.^43^ For instance, Pettersson *et al*.^38^reviewed large cohort of 68 NSCLC patients treated 45 Gy in 3 fractions of lung SBRT. Among the 33 patients with complete clinical and radiographic follow up exceeding 15 months, 13 rib fractures were found in seven patients. In their study, the logistic dose‐response curve exhibited that the risk of radiation‐induced rib fractures following lung SBRT treatments were related to the dose to 2 cc of the rib. For a median follow up of 29 months, they showed that the 2 cc of rib receiving total 27.3 Gy in 3 fractions had 5% chance of rib fracture. In the current study, our h‐DCA with 6X‐FFF beam provided lower dose tolerances to all OAR (including rib, lung, and skin) compared to clinical VMAT plans and all OAR dose metrics were well below the RTOG criteria. Therefore, we do not anticipate any acute or late toxicity. However, clinical follow up of tumor local‐control and treatment‐related toxicities of these patients is necessary.

In summary, each h‐DCA plan was thoroughly evaluated using the dosimetric and treatment delivery parameters listed in Tables 2 and 3. All parameters were deemed acceptable for both h‐DCA and clinical VMAT plans per SBRT protocol requirements suggesting that h‐DCA plans are dosimetrically equivalent to clinical VMAT plans. With h‐DCA, faster treatment delivery is possible, potentially benefiting patients who cannot lie flat in the treatment position for a long period of time. In addition, h‐DCA overcomes concerns over the accuracy of the dose calculation and delivery errors for small fields (beamlets) in areas of tissue interfaces and potentially minimizes the MLC interplay effect (with no MLC modulation through the target) as demonstrated in phantom QA measurement (see Table 3). Moreover, h‐DCA could allow for real‐time target verification (with no MLC modulation through the target, it allows for imaging treatment fields during treatment, if desired) and also eliminates patient‐specific VMAT QA–potentially offering cost‐effective, same or next day SBRT treatments to lung lesions. This technique can be easily adopted to other diseases sites (including hypofractionated centrally located lung lesions, stereotactic treatment of brain or abdominal/pelvis lesions including liver SBRT) and small radiotherapy clinics with less extensive physics or machine support for SBRT treatments. However, larger lung lesions seated near the critical structures or re‐irradiation patients can potentially benefit with highly optimized IMRT/VMAT plans.^44^, ^45^, ^46^ Future work includes adding a few field‐in‐field control points into those static beams to further improve our h‐DCA plan quality. Due to decreased MU and BOT with h‐DCA planning, deep inspiration breath‐hold lung SBRT planning may be of value in future investigations.

## 5 CONCLUSION

A simple, yet clinically useful h‐DCA planning technique was devised for lung SBRT treatments. The h‐DCA technique minimizes small‐field dosimetry errors and MLC interplay effects, resulting in enhanced target coverage by improving the target dose heterogeneities in the tumor center (characteristic of FFF‐beams). The h‐DCA simplifies treatment planning and delivery by significantly reducing treatment planning time and BOT when compared to clinical VMAT lung SBRT plans. Furthermore, due to no MLC modulation over the target, h‐DCA allows for real‐time target verification using trigger imaging and eliminates patient‐specific QA. Overall, treatment planning time for the h‐DCA technique was about an hour, which can potentially allow for cost‐effective same or next day SBRT treatments to lung lesion. Another major advantage of the h‐DCA technique is that it can be easily adopted to small community sites with less extensive physics and machine resources; potentially expanding SBRT programs to satellite clinics. The h‐DCA planning can be easily adopted to other disease sites such as stereotactic treatment of brain or any abdominal/pelvis lesions such as liver, pancreas, or adrenal glands SBRT.

## Conflict of interests

The author have no conflict of interests to disclose.
